# Molecular classification of solid tumours: towards pathway-driven therapeutics

**DOI:** 10.1038/sj.bjc.6605031

**Published:** 2009-04-14

**Authors:** C Swanton, C Caldas

**Affiliations:** 1 Translational Cancer Therapeutics Laboratory, Cancer Research UK London Research Institute, 44 Lincoln's Inn Fields, London WC2A 3PX, UK; 2 Department of Medicine, Royal Marsden Hospital, Downs Road, Sutton, SM2 5PT, UK; 3 Department of Oncology, University of Cambridge Functional Breast Cancer Genomics Laboratory, Cancer Research UK Cambridge Research Institute, Li Ka-Shing Centre, Robinson Way, Cambridge CB2 0RE

**Keywords:** genomic instability, chromosomal instability, drug resistance, gene expression, DNA copy number analysis, microRNA

## Abstract

The last decade has witnessed unprecedented developments in the genetic and epigenetic analyses of solid tumours. Transcriptional and DNA copy-number studies have improved our understanding and classification of solid tumours and highlighted the patterns of genomic aberrations associated with outcome. The identification of altered transcriptional and translational silencing by microRNAs and epigenetic modification by methylation in tumours has showed a layer of additional intricacy to the regulation of gene expression in different tumour types. The advent of massive parallel sequencing has allowed whole cancer genomes to be sequenced with extraordinary speed and accuracy providing insight into the bewildering complexity of gene mutations present in solid tumours. Functional genomic studies using RNA interference-screening tools promises to improve the classification of solid tumours by probing the relevance of each gene to tumour phenotype. In this review, we discuss how these studies have contributed to solid tumour classification and why such studies are central to the future of oncology. We suggest that these developments are gradually leading to a change in emphasis of early clinical trials to a therapeutic model guided by the molecular classification of tumours. The investigation of drug efficacy later in development is beginning to rely on patient selection defined by predictive molecular criteria that complement solid tumour classification based on anatomic site.

## Background

Therapeutic decision-making in oncology after surgical resection of the tumour (adjuvant treatment) is based on an assessment of the risk of tumour relapse. Risk profiling is a complex and often imprecise task that has focussed on the clinical and histopathological features of the tumour. Molecular profiling of gene expression over the last decade has shown that heterogeneity in outcome and survival in cancer can be explained, in part, by genomic variation within the primary tumour (Brenton *et al*, 2005).

**Figure 1:**
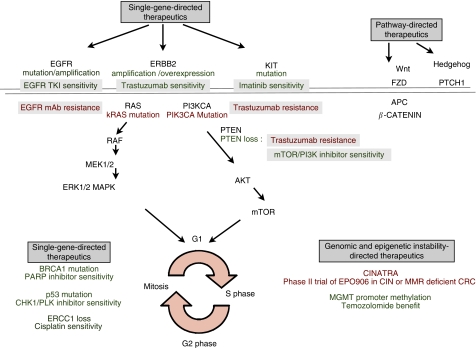
Classification of defined genetic abnormalities in solid tumours to optimise tumour response. Figure shows the three methods through which improved molecular classification of solid tumours is challenging the traditional approach to cytotoxic delivery (green font indicates theoretical or proven sensitivity and red font indicates resistance based on molecular aberration). Mutations (EGFR) or amplification (HER2) guides the use of EGFR tyrosine kinase inhibitors in NSCLC and breast cancer, respectively. Assessing whether genomic instability is an exploitable phenotype is currently under investigation (CINATRA: chromosomal instability and anti-tubulin response assessment) and new approaches evolving from molecular analysis of solid tumours may be directed towards activated pathways in solid tumours through the attenuation of Wnt and Hedgehog signalling. CIN=chromosomal instability, MMR=mismatch repair, CRC=colorectal cancer, mAb=monoclonal antibody, EGFR=epidermal growth factor receptor.

Recent developments in tumour classification have shown that although the genetic mutations that occur in solid tumours may be heterogeneous and complex, mutations often occur in genes that function in common cell signalling pathways that may render them sensitive to targeted approaches (Figure 1). The implications for the management of solid tumours are profound. Not only will these strategies improve the classification within each tumour type, but they will also guide the development of therapeutic approaches to limit tumour growth and contribute to the delivery of personalised medical care (van't Veer and Bernards, 2008). These developments will lead to the exploitation of specific signalling pathways in individual tumours that will cross organ-specific boundaries to improve patient outcome and survival. Such progress promises to drive the identification of predictive models of drug response enabling drug delivery governed by the genomic heterogeneity specific to each tumour. Predictive models are those used to predict response to a particular therapy and prognostic models are those used to predict clinical outcome independently of therapy.

We suggest that improved classification of solid tumours will contribute to the development of therapeutic strategies guided either by aberrations in single genes, or by the activation of specific signalling pathways, or by patterns of tumour genomic instability (Figure 1).

## Gene expression profiling and DNA copy-number analysis

Improved molecular classification of solid tumours is essential to identify biomarkers, which reflect the molecular mechanisms functionally involved in tumour-type specific survival, drug resistance, tumour relapse and patient outcome in a highly sensitive and specific manner. Current molecular classification models have not been proven to act as independent markers of disease outcome in comparison to validated methods and have frequently not adhered to strict guidelines required for biomarker validation (McShane *et al*, 2005). Ultimately, refined molecular models classifying solid tumours will lead to the identification of new drug targets and patients at low risk of relapse who will not benefit from adjuvant chemotherapy (Swanton *et al*, 2008).

There have been several issues raised with molecular classification and forecasting strategies in cancer medicine regarding the reproducibility of predictive expression signatures derived from relatively small studies (Brenton *et al*, 2005; Ein-Dor *et al*, 2006). Studies used to derive gene expression signatures predictive of outcome or treatment response have frequently been derived from small and clinically heterogeneous patient series with validation and training tumours originating from the same patient cohorts. The capacity of hypothesis-generating microarray studies to lead to biased predictive models that ‘overfit’ a list of genes to the cohort under study, that may not validate in truly independent and unrelated clinical cohorts, is well documented. As a result, recent research has focussed on a refinement of predictive classifiers from well-characterised, rather than clinically heterogeneous, patient cohorts.

One such approach has been published recently in a homogeneous group of patients with adenocarcinoma of the lung of defined clinical stage to attempt to define new prognostic models suitable for clinical application across multiple hospital sites (Shedden *et al*, 2008). The authors standardised pathological assessment, collection of tumours and clinical information from each institution involved in the study at the outset. Two-blinded external validation cohorts were used to test their prognostic methods, unlike many earlier studies that have tended to divide a single cohort from one study into a training and validation set. Higher expression of genes involved in cell-cycle progression and chromosome segregation correlated with poorer outcome. The authors noted the improved performance of their models when clinical and gene expression data were combined.

Alternative approaches, include retrospective analyses of tissue from clinical trials. The Trial Assigning IndividuaLized Options for Treatment (Rx), (TAILORx: http://www.cancer.gov/clinicaltrials/ECOG-PACCT-1) is currently validating one such 21 gene signature by RT–PCR, developed from an analysis of three breast cancer studies in 447 patients, to guide risk stratification among patients with node-negative oestrogen receptor (ER)-positive breast cancer treated with tamoxifen (Paik *et al*, 2004; and for review see Swanton *et al*, 2008).

Traditional prognostic classifiers from heterogeneous clinicopathological patient groups have failed to provide a robust signature predictive of outcome in ER-negative breast cancer for which prognostic signals seem to be much weaker (Teschendorff *et al*, 2007). Profiling studies using a novel statistical approach that may reduce the false discovery rate have shown that ER-negative breast cancer is divided into four main subtypes of disease and that heterogeneity in outcome relates to the expression of an immune response gene expression set that seems to be independent of lymphocyte recruitment (Teschendorff *et al*, 2007).

Changes in DNA copy number have been shown to drive a high proportion of changes at the mRNA level and outcome stratification in breast cancer can be improved by the parallel assessment of gene expression and change in copy number (Chin *et al*, 2006). Studies of DNA copy number alterations with expression data in 171 breast cancers of relatively small size and low Nottingham Prognostic Index (reflective of breast cancer demographics in the modern screening era) have been published recently (Chin *et al*, 2007). This work highlighted that breast cancer is considerably more heterogeneous than earlier studies had portrayed (Chin *et al*, 2006) and showed the existence of a low genomic instability index (defined as the fraction of the genome altered) tumour cohort consisting disproportionately of ER-negative and basal samples. Two other clusters consisted of intermediate/high grade tumours characterised by high genomic instability index. Therefore, ER-negative tumours seem to have distinct clusters defined by high or low genomic instability index. Intriguingly, unlike ER-positive tumours for which genomic instability index correlates with clinical outcome, no such correlation was observed in ER-negative tumours.

## Chromosomal instability classifies solid tumours into a poor prognostic category

There is now good pre-clinical evidence to suggest that distinct patterns of genomic instability, as opposed to defined aberrations in signalling pathways discussed later, may affect the therapeutic response.

Chromosomal instability (CIN) is associated with poorer outcome in many solid tumours including breast and colon cancer (Furuya *et al*, 2000; Risques *et al*, 2003; Kronenwett *et al*, 2004; Carter *et al*, 2006; Walther *et al*, 2008). Recently, a gene expression signature associated with CIN and the combined level of tumour chromosomal aberrations, termed total functional aneuploidy, predicted poorer outcome in six cancer types (Carter *et al*, 2006). The expression of the CIN signature was higher in metastatic samples compared with primary tumours and was able to classify grade 1 and 2 breast cancers according to outcome.

Rapid acquisition of multidrug resistance is frequently witnessed after the exposure of solid tumours to consecutive ‘non-cross resistant’ chemotherapy regimens, with diminished patient benefit as lines of treatment progress. Aneuploid cells acquire multidrug resistance at a higher rate than diploid cells that may be catalysed by chromosome reassortments at each mitosis (Duesberg *et al*, 2000). Chromosome missegregation may promote an evolutionary benefit within the solid tumour mass by altering gene dosage across the tumour population as evidenced by the transcriptional changes induced by single-cell chromosome transfer experiments (Upender *et al*, 2004). A compelling hypothesis generated from these observations is that the selection of a drug resistance gene encoded on a missegregated chromosome after chemotherapy exposure is associated with ‘multiple unselected phenotypes’ induced by genes encoded on the same chromosome that may promote resistance to unrelated drug compounds (Duesberg *et al*, 2000).

CIN may refer to both structural DNA copy-number alterations and numerical changes of whole chromosomes. Evidence suggests that CIN may be an exploitable phenotype and that cytotoxic compounds exist that may have preferential activity in cells with distinct patterns of genomic instability (Roschke and Kirsch, 2005). Conversely, functional genomic analysis has identified that numerically CIN tumour cells may be relatively resistant to microtubule stabilising agents owing to similarities between pathways regulating the separation of chromosomes at mitosis and response to taxanes (Swanton *et al*, 2007). In contrast, our evidence suggests that a near diploid stable karyotype (frequently observed in mismatch repair deficient, microsatellite-unstable colorectal cancers) is associated with sensitivity to mitotic inhibitors, such as taxanes and kinesin 5 inhibitors. This work has led to the initiation of a phase II clinical trial of Epothilone 906 in colorectal cancer. This trial, called CINATRA (Chromosomal Instability and Anti-Tubulin Response Assessment) aims to assess whether patients with MMR deficient, karyotypically stable colorectal cancers harbour disease, which is intrinsically more sensitive to microtubule-stabilising agents than the CIN tumours (Swanton *et al*, 2006). The challenge over the next decade will be to identify therapeutic strategies to limit chromosome missegregation and CIN and exploit weaknesses inherent to tumour karyotype as a targetable phenotype.

## Functional genomic tumour classification and therapeutic selection

Studies have shown that drugs with similar mechanisms of action share common gene expression signatures that may play a functional role in drug response (Lamb *et al*, 2006). This has led to the concept that genetic heterogeneity within the primary tumour may influence drug response and provoked efforts to classify tumours by drug sensitivity status. Unfortunately, small datasets, from which the early studies were derived, have not yielded gene signatures of sufficient power to predict drug sensitivity (Sorlie *et al*, 2006). Efforts to improve on these signatures by combining cell-line gene expression analysis with matched drug sensitivity data to classify sensitivity to combination chemotherapy *in vivo* have been published (Potti *et al*, 2006; Bonnefoi *et al*, 2007). The negative predictive value of this method may be sufficiently robust to enable the selection of patients with multidrug resistant disease for novel therapeutic clinical trials.

The identification of genes functionally involved in drug response using RNA interference-screening techniques has started to yield information enabling the genetic dissection of tumour gene expression. RNA interference screening was used recently to determine that PI3K pathway activation, through PTEN loss and/or PI3KCA mutation, was the main regulator of trastuzumab resistance (Berns *et al*, 2007) indicating that these patients may derive more benefit from treatment with AKT or mTOR inhibitors.

## Tumour classification based on miRNA analysis

MicroRNAs (miRNAs) are small non-coding RNAs that negatively regulate gene expression through the inhibition of mRNA translation or the initiation of mRNA degradation. The tissue-specific expression of miRNAs has provoked intense investigation to identify whether these non-coding RNAs can reflect signatures of tumour origin, prognosis and outcome. In one of the first investigations of 217 miRNAs in 334 tumour and normal tissue samples, miRNA expression profiling segregated tumours by developmental origin and had the capacity to correctly identify the tissue of origin of poorly differentiated tumours, in contrast to an mRNA-based tissue classifier (Lu *et al*, 2005). In contrast to mRNA profiling, which does not consistently differentiate between tumour and normal tissue, the majority of miRNAs studied had lower expression in tumour compared with normal tissue. The authors suggest that tumour-specific miRNA repression results from differences in the differentiation status between tumour and normal tissue and propose that a signature of 200 miRNAs may be sufficient to classify human cancer. In agreement with these data, Rosetta Genomics showed the potential of miRNA profiling to define the origin of carcinoma of unknown primary and accurately classify tumour type (Rosenfeld *et al*, 2008).

In a breast cancer specific study comparing miRNA to mRNA-expression profiles, we have showed that several miRNAs are differentially expressed between the Luminal A/B, Her2+, basal and normal-like breast cancers (Blenkiron *et al*, 2007). This study shows that alterations in miRNA expression are complex and may relate to genomic loss or gain, changes in primary transcription and miRNA biogenesis. We did not detect significant enrichment for down- or up-regulated predicted target mRNAs and suggest that the predominant activity of miRNA influence lies in mRNA translational control.

## miRNA expression and prognostication

Expression of miRNAs may offer prognostic and predictive information in several solid tumours. Yu *et al* (2008) presented data showing that a signature of five miRNAs can predict treatment outcome in NSCLC and serves as an independent predictor of recurrence free and overall survival. Four miRNAs; miR-7, miR-128a, miR-210 and miR-516-3p are associated with tumour aggressiveness in ER-positive, lymph-node negative breast cancer. Expression of miR-210 was associated with early relapse in ER-negative, lymph-node negative disease and in a cohort of 69 triple-negative breast cancers (Foekens *et al*, 2008). The potential prognostic role of miR-210 in this disease has been supported in a separate study, which showed the induction of miR-210 by tumour hypoxia in a HIF1a-dependent manner (Camps *et al*, 2008).

If the association of miRNAs with prognosis can be shown to be directly causative, they may represent excellent therapeutic targets because of their potential ability to influence multiple biological processes by altering the expression and translation of many mRNAs. In this regard, several miRNAs have now been attributed with direct roles in tumour invasion, tumour growth and motility and in the activation or repression of cancer survival signalling and angiogenic pathways (Ovcharenko *et al*, 2007; Fish *et al*, 2008; Kefas *et al*, 2008; Lujambio *et al*, 2008).

## Cancer genome sequencing studies

Sequencing analysis of the human genome has enabled benchmarks to be set, by which cancer genomes can be compared. Developments in bioinformatics tools and sequencing platforms have led to the publication of several genome-wide sequencing studies in solid tumours. In 2006, Velculescu *et al*. (Sjoblom *et al*, 2006) presented sequencing results for 13 023 genes in 11 breast and 11 colorectal cancers. Following stringent validation procedures they identified 365 mutations in 236 genes. This study showed that each tumour harboured mutations in ∼90 genes that displayed significant heterogeneity between tumour types.

This group and others have provided similar genomic analysis of glioblastoma multiforme and pancreatic cancer (Jones *et al*, 2008; McLendon *et al*, 2008; Parsons *et al*, 2008). After sequencing of 20 661 genes, 63 genetic alterations were found on average in each pancreatic cancer belonging to a set of 12 signalling pathways altered in greater than two-thirds of tumours (Jones *et al*, 2008). The authors speculate that the lower somatic mutation rate in pancreatic cancer compared with breast or colorectal cancer may be explained by a requirement for fewer divisions in the initiation of pancreatic neoplasia compared with breast or colorectal cancer.

Glioblastoma multiforme (GBM) sequencing analysis has shown recurrent mutations in isocitrate dehydrogenase 1 (IDH1) in 12% of patients (Parsons *et al*, 2008). These mutations seemed to occur in younger patients and most patients with secondary GBMs. Tumours harbouring these mutations seem to be associated with an increase in overall survival of patients. Pathways altered in these tumours were similar to those in pancreatic, breast and colorectal cancer, although some GBM-specific pathways incorporating ion-channels and pathways involved in transmission of synaptic or neural signals and axonal guidance were noted.

The Cancer Genome Atlas Research Network recently published data of a similar analysis in GBM-combining gene expression profiling, DNA copy-number analysis with methylation and nucleotide sequencing analysis (McLendon *et al*, 2008). These data show a connection between MGMT promoter methylation and the hypermutator phenotype in temozolomide-treated GBM. The MGMT promoter methylation results in a failure to repair alkylated guanine residues resulting from treatment. Unrepaired alkylated guanines initiate repetitive cycles of mismatch repair triggering cell death. The dependence of cell death on a competent MMR pathway initiates a strong selective pressure for inactivation of the MMR pathway and the resultant hypermutator phenotype. This is one of the first studies to show the emergence of genomic instability resulting from a selection pressure driven by chemotherapy and has major clinical implications regarding the use of temozolomide and the development of new drugs in this disease.

## Conclusions and perspectives

Classification studies in solid tumours have showed the complex heterogeneity of gene and miRNA expression that may account for the unpredictable clinical behaviour and response to therapy in solid tumours, exacerbated further by underlying tumour genomic instability. We propose a model, (Figure 1) whereby improved molecular classification of solid tumours is in the process of directing therapeutics guided by the status of single genes within the tumour (mutation/deletion/amplification or loss), the activation of specific signalling pathways and the genomic instability and epigenetic status of the tumour.

Clinical trials investigating activity of EGFR-directed therapies using kRAS and EGFR receptor mutation status as biomarkers of response have altered the landscape of targeted therapy use. Clinical strategies to maximise response to Her2-targeted agents in tumours with PI3KCA mutations and/or PTEN loss are being considered based on knowledge of downstream signalling pathways. The specific targeting of a distinct pattern of genomic instability is the subject of investigation in the phase II trial CINATRA discussed in this paper.

Finally, although most genes are only mutated at low frequency in each tumour type, the identification of functional gene groups belonging to the same signalling pathway, may provide opportunities for therapeutic exploitation. For example, all pancreatic cancers analysed had gene alterations in the Wnt and Hedgehog signalling pathways. Therefore, although a large number of genes are mutated in solid tumours, the number of pathways through which they function is relatively small. These studies provide a compelling focus for pathway directed, rather than traditional tumour-type specific interventions.

## References

[bib1] Berns K, Horlings HM, Hennessy BT, Madiredjo M, Hijmans EM, Beelen K, Linn SC, Gonzalez-Angulo AM, Stemke-Hale K, Hauptmann M, Beijersbergen RL, Mills GB, van de Vijver MJ, Bernards R (2007) A functional genetic approach identifies the PI3K pathway as a major determinant of trastuzumab resistance in breast cancer. Cancer Cell 12: 395–402 17936563 10.1016/j.ccr.2007.08.030

[bib2] Blenkiron C, Goldstein LD, Thorne NP, Spiteri I, Chin SF, Dunning MJ, Barbosa-Morais NL, Teschendorff AE, Green AR, Ellis IO, Tavare S, Caldas C, Miska EA (2007) MicroRNA expression profiling of human breast cancer identifies new markers of tumor subtype. Genome Biol 8: R214 17922911 10.1186/gb-2007-8-10-r214 PMC2246288

[bib3] Bonnefoi H, Potti A, Delorenzi M, Mauriac L, Campone M, Tubiana-Hulin M, Petit T, Rouanet P, Jassem J, Blot E, Becette V, Farmer P, Andre S, Acharya CR, Mukherjee S, Cameron D, Bergh J, Nevins JR, Iggo RD (2007) Validation of gene signatures that predict the response of breast cancer to neoadjuvant chemotherapy: a substudy of the EORTC 10994/BIG 00-01 clinical trial. Lancet Oncol 8: 1071–1078 18024211 10.1016/S1470-2045(07)70345-5

[bib4] Brenton JD, Carey LA, Ahmed AA, Caldas C (2005) Molecular classification and molecular forecasting of breast cancer: ready for clinical application? J Clin Oncol 23: 7350–7360 16145060 10.1200/JCO.2005.03.3845

[bib5] Camps C, Buffa FM, Colella S, Moore J, Sotiriou C, Sheldon H, Harris AL, Gleadle JM, Ragoussis J (2008) hsa-miR-210 Is induced by hypoxia and is an independent prognostic factor in breast cancer. Clin Cancer Res 14: 1340–1348 18316553 10.1158/1078-0432.CCR-07-1755

[bib6] Carter SL, Eklund AC, Kohane IS, Harris LN, Szallasi Z (2006) A signature of chromosomal instability inferred from gene expression profiles predicts clinical outcome in multiple human cancers. Nat Genet 38: 1043–1048 16921376 10.1038/ng1861

[bib7] Chin K, DeVries S, Fridlyand J, Spellman PT, Roydasgupta R, Kuo WL, Lapuk A, Neve RM, Qian Z, Ryder T, Chen F, Feiler H, Tokuyasu T, Kingsley C, Dairkee S, Meng Z, Chew K, Pinkel D, Jain A, Ljung BM, Esserman L, Albertson DG, Waldman FM, Gray JW (2006) Genomic and transcriptional aberrations linked to breast cancer pathophysiologies. Cancer Cell 10: 529–541 17157792 10.1016/j.ccr.2006.10.009

[bib8] Chin SF, Teschendorff AE, Marioni JC, Wang Y, Barbosa-Morais NL, Thorne NP, Costa JL, Pinder SE, van de Wiel MA, Green AR, Ellis IO, Porter PL, Tavare S, Brenton JD, Ylstra B, Caldas C (2007) High-resolution aCGH and expression profiling identifies a novel genomic subtype of ER negative breast cancer. Genome Biol 8: R215 17925008 10.1186/gb-2007-8-10-r215 PMC2246289

[bib9] Duesberg P, Stindl R, Hehlmann R (2000) Explaining the high mutation rates of cancer cells to drug and multidrug resistance by chromosome reassortments that are catalysed by aneuploidy. Proc Natl Acad Sci USA 97: 14295–14300 11121035 10.1073/pnas.97.26.14295 PMC18912

[bib10] Ein-Dor L, Zuk O, Domany E (2006) Thousands of samples are needed to generate a robust gene list for predicting outcome in cancer. Proc Natl Acad Sci USA 103: 5923–5928 16585533 10.1073/pnas.0601231103 PMC1458674

[bib11] Fish JE, Santoro MM, Morton SU, Yu S, Yeh RF, Wythe JD, Ivey KN, Bruneau BG, Stainier DY, Srivastava D (2008) miR-126 regulates angiogenic signaling and vascular integrity. Dev Cell 15: 272–284 18694566 10.1016/j.devcel.2008.07.008 PMC2604134

[bib12] Foekens JA, Sieuwerts AM, Smid M, Look MP, de Weerd V, Boersma AW, Klijn JG, Wiemer EA, Martens JW (2008) Four miRNAs associated with aggressiveness of lymph node-negative, estrogen receptor-positive human breast cancer. Proc Natl Acad Sci USA 105: 13021–13026 18755890 10.1073/pnas.0803304105 PMC2529088

[bib13] Furuya T, Uchiyama T, Murakami T, Adachi A, Kawauchi S, Oga A, Hirano T, Sasaki K (2000) Relationship between chromosomal instability and intratumoral regional DNA ploidy heterogeneity in primary gastric cancers. Clin Cancer Res 6: 2815–2820 10914729

[bib14] Jones S, Zhang X, Parsons DW, Lin JC, Leary RJ, Angenendt P, Mankoo P, Carter H, Kamiyama H, Jimeno A, Hong SM, Fu B, Lin MT, Calhoun ES, Kamiyama M, Walter K, Nikolskaya T, Nikolsky Y, Hartigan J, Smith DR, Hidalgo M, Leach SD, Klein AP, Jaffee EM, Goggins M, Maitra A, Iacobuzio-Donahue C, Eshleman JR, Kern SE, Hruban RH, Karchin R, Papadopoulos N, Parmigiani G, Vogelstein B, Velculescu VE, Kinzler KW (2008) Core signaling pathways in human pancreatic cancers revealed by global genomic analyses. Science 321: 1801–1806 18772397 10.1126/science.1164368 PMC2848990

[bib15] Kefas B, Godlewski J, Comeau L, Li Y, Abounader R, Hawkinson M, Lee J, Fine H, Chiocca EA, Lawler S, Purow B (2008) microRNA-7 inhibits the epidermal growth factor receptor and the Akt pathway and is down-regulated in glioblastoma. Cancer Res 68: 3566–3572 18483236 10.1158/0008-5472.CAN-07-6639

[bib16] Kronenwett U, Huwendiek S, Ostring C, Portwood N, Roblick UJ, Pawitan Y, Alaiya A, Sennerstam R, Zetterberg A, Auer G (2004) Improved grading of breast adenocarcinomas based on genomic instability. Cancer Res 64: 904–909 14871819 10.1158/0008-5472.can-03-2451

[bib17] Lamb J, Crawford ED, Peck D, Modell JW, Blat IC, Wrobel MJ, Lerner J, Brunet JP, Subramanian A, Ross KN, Reich M, Hieronymus H, Wei G, Armstrong SA, Haggarty SJ, Clemons PA, Wei R, Carr SA, Lander ES, Golub TR (2006) The Connectivity Map: using gene-expression signatures to connect small molecules, genes, and disease. Science 313: 1929–1935 17008526 10.1126/science.1132939

[bib18] Lu J, Getz G, Miska EA, Alvarez-Saavedra E, Lamb J, Peck D, Sweet-Cordero A, Ebert BL, Mak RH, Ferrando AA, Downing JR, Jacks T, Horvitz HR, Golub TR (2005) MicroRNA expression profiles classify human cancers. Nature 435: 834–838 15944708 10.1038/nature03702

[bib19] Lujambio A, Calin GA, Villanueva A, Ropero S, Sanchez-Cespedes M, Blanco D, Montuenga LM, Rossi S, Nicoloso MS, Faller WJ, Gallagher WM, Eccles SA, Croce CM, Esteller M (2008) A microRNA DNA methylation signature for human cancer metastasis. Proc Natl Acad Sci USA 105: 13556–13561 18768788 10.1073/pnas.0803055105 PMC2528872

[bib20] McLendon R, Friedman A, Bigner D, Van Meir EG, Brat DJ, Mastrogianakis M, Olson JJ, Mikkelsen T, Lehman N, Aldape K, Alfred Yung WK, Bogler O, Vandenberg S, Berger M, Prados M, Muzny D, Morgan M, Scherer S, Sabo A, Nazareth L, Lewis L, Hall O, Zhu Y, Ren Y, Alvi O, Yao J, Hawes A, Jhangiani S, Fowler G, San Lucas A, Kovar C, Cree A, Dinh H, Santibanez J, Joshi V, Gonzalez-Garay ML, Miller CA, Milosavljevic A, Donehower L, Wheeler DA, Gibbs RA, Cibulskis K, Sougnez C, Fennell T, Mahan S, Wilkinson J, Ziaugra L, Onofrio R, Bloom T, Nicol R, Ardlie K, Baldwin J, Gabriel S, Lander ES, Ding L, Fulton RS, McLellan MD, Wallis J, Larson DE, Shi X, Abbott R, Fulton L, Chen K, Koboldt DC, Wendl MC, Meyer R, Tang Y, Lin L, Osborne JR, Dunford-Shore BH, Miner TL, Delehaunty K, Markovic C, Swift G, Courtney W, Pohl C, Abbott S, Hawkins A, Leong S, Haipek C, Schmidt H, Wiechert M, Vickery T, Scott S, Dooling DJ, Chinwalla A, Weinstock GM, Mardis ER, Wilson RK, Getz G, Winckler W, Verhaak RG, Lawrence MS, O'Kelly M, Robinson J, Alexe G, Beroukhim R, Carter S, Chiang D, Gould J, Gupta S, Korn J, Mermel C, Mesirov J, Monti S, Nguyen H, Parkin M, Reich M, Stransky N, Weir BA, Garraway L, Golub T, Meyerson M, Chin L, Protopopov A, Zhang J, Perna I, Aronson S, Sathiamoorthy N, Ren G, Yao J, Wiedemeyer WR, Kim H, Won Kong S, Xiao Y, Kohane IS, Seidman J, Park PJ, Kucherlapati R, Laird PW, Cope L, Herman JG, Weisenberger DJ, Pan F, Van Den Berg D, Van Neste L, Mi Yi J, Schuebel KE, Baylin SB, Absher DM, Li JZ, Southwick A, Brady S, Aggarwal A, Chung T, Sherlock G, Brooks JD, Myers RM, Spellman PT, Purdom E, Jakkula LR, Lapuk AV, Marr H, Dorton S, Gi Choi Y, Han J, Ray A, Wang V, Durinck S, Robinson M, Wang NJ, Vranizan K, Peng V, Van Name E, Fontenay GV, Ngai J, Conboy JG, Parvin B, Feiler HS,Speed TP, Gray JW, Brennan C, Socci ND, Olshen A, Taylor BS, Lash A, Schultz N, Reva B, Antipin Y, Stukalov A, Gross B, Cerami E, Qing Wang W, Qin LX, Seshan VE, Villafania L, Cavatore M, Borsu L, Viale A, Gerald W, Sander C, Ladanyi M, Perou CM, Neil Hayes D, Topal MD, Hoadley KA, Qi Y, Balu S, Shi Y, Wu J, Penny R, Bittner M, Shelton T, Lenkiewicz E, Morris S, Beasley D, Sanders S, Kahn A, Sfeir R, Chen J, Nassau D, Feng L, Hickey E, Zhang J, Weinstein JN, Barker A, Gerhard DS, Vockley J, Compton C, Vaught J, Fielding P, Ferguson ML, Schaefer C, Madhavan S, Buetow KH, Collins F, Good P, Guyer M, Ozenberger B, Peterson J, Thomson E (2008) Comprehensive genomic characterization defines human glioblastoma genes and core pathways. Nature 455: 1061–1068 18772890 10.1038/nature07385 PMC2671642

[bib21] McShane LM, Altman DG, Sauerbrei W, Taube SE, Gion M, Clark GM (2005) REporting recommendations for tumour MARKer prognostic studies (REMARK). Br J Cancer 93: 387–391 16106245 10.1038/sj.bjc.6602678 PMC2361579

[bib22] Ovcharenko D, Kelnar K, Johnson C, Leng N, Brown D (2007) Genome-scale microRNA and small interfering RNA screens identify small RNA modulators of TRAIL-induced apoptosis pathway. Cancer Res 67: 10782–10788 18006822 10.1158/0008-5472.CAN-07-1484

[bib23] Paik S, Shak S, Tang G, Kim C, Baker J, Cronin M, Baehner FL, Walker MG, Watson D, Park T, Hiller W, Fisher ER, Wickerham DL, Bryant J, Wolmark N (2004) A multigene assay to predict recurrence of tamoxifen-treated, node-negative breast cancer. N Engl J Med 351: 2817–2826 15591335 10.1056/NEJMoa041588

[bib24] Parsons DW, Jones S, Zhang X, Lin JC, Leary RJ, Angenendt P, Mankoo P, Carter H, Siu IM, Gallia GL, Olivi A, McLendon R, Rasheed BA, Keir S, Nikolskaya T, Nikolsky Y, Busam DA, Tekleab H, Diaz Jr LA, Hartigan J, Smith DR, Strausberg RL, Marie SK, Shinjo SM, Yan H, Riggins GJ, Bigner DD, Karchin R, Papadopoulos N, Parmigiani G, Vogelstein B, Velculescu VE, Kinzler KW (2008) An integrated genomic analysis of human glioblastoma multiforme. Science 321: 1807–1812 18772396 10.1126/science.1164382 PMC2820389

[bib25] Potti A, Dressman HK, Bild A, Riedel RF, Chan G, Sayer R, Cragun J, Cottrill H, Kelley MJ, Petersen R, Harpole D, Marks J, Berchuck A, Ginsburg GS, Febbo P, Lancaster J, Nevins JR (2006) Genomic signatures to guide the use of chemotherapeutics. Nat Med 12: 1294–1300 17057710 10.1038/nm1491

[bib26] Risques RA, Moreno V, Ribas M, Marcuello E, Capella G, Peinado MA (2003) Genetic pathways and genome-wide determinants of clinical outcome in colorectal cancer. Cancer Res 63: 7206–7214 14612515

[bib27] Roschke AV, Kirsch IR (2005) Targeting cancer cells by exploiting karyotypic complexity and chromosomal instability. Cell Cycle 4: 679–682 15846096 10.4161/cc.4.5.1687

[bib28] Rosenfeld N, Aharonov R, Meiri E, Rosenwald S, Spector Y, Zepeniuk M, Benjamin H, Shabes N, Tabak S, Levy A, Lebanony D, Goren Y, Silberschein E, Targan N, Ben-Ari A, Gilad S, Sion-Vardy N, Tobar A, Feinmesser M, Kharenko O, Nativ O, Nass D, Perelman M, Yosepovich A, Shalmon B, Polak-Charcon S, Fridman E, Avniel A, Bentwich I, Bentwich Z, Cohen D, Chajut A, Barshack I (2008) MicroRNAs accurately identify cancer tissue origin. Nat Biotechnol 26: 462–469 18362881 10.1038/nbt1392

[bib29] Shedden K, Taylor JM, Enkemann SA, Tsao MS, Yeatman TJ, Gerald WL, Eschrich S, Jurisica I, Giordano TJ, Misek DE, Chang AC, Zhu CQ, Strumpf D, Hanash S, Shepherd FA, Ding K, Seymour L, Naoki K, Pennell N, Weir B, Verhaak R, Ladd-Acosta C, Golub T, Gruidl M, Sharma A, Szoke J, Zakowski M, Rusch V, Kris M, Viale A, Motoi N, Travis W, Conley B, Seshan VE, Meyerson M, Kuick R, Dobbin KK, Lively T, Jacobson JW, Beer DG (2008) Gene expression-based survival prediction in lung adenocarcinoma: a multi-site, blinded validation study. Nat Med 14: 822–827 18641660 10.1038/nm.1790 PMC2667337

[bib30] Sjoblom T, Jones S, Wood LD, Parsons DW, Lin J, Barber TD, Mandelker D, Leary RJ, Ptak J, Silliman N, Szabo S, Buckhaults P, Farrell C, Meeh P, Markowitz SD, Willis J, Dawson D, Willson JK, Gazdar AF, Hartigan J, Wu L, Liu C, Parmigiani G, Park BH, Bachman KE, Papadopoulos N, Vogelstein B, Kinzler KW, Velculescu VE (2006) The consensus coding sequences of human breast and colorectal cancers. Science 314: 268–274 16959974 10.1126/science.1133427

[bib31] Sorlie T, Perou CM, Fan C, Geisler S, Aas T, Nobel A, Anker G, Akslen LA, Botstein D, Borresen-Dale AL, Lonning PE (2006) Gene expression profiles do not consistently predict the clinical treatment response in locally advanced breast cancer. Mol Cancer Ther 5: 2914–2918 17121939 10.1158/1535-7163.MCT-06-0126

[bib32] Swanton C, Marani M, Pardo O, Warne PH, Kelly G, Sahai E, Elustondo F, Chang J, Temple J, Ahmed AA, Brenton JD, Downward J, Nicke B (2007) Regulators of mitotic arrest and ceramide metabolism are determinants of sensitivity to paclitaxel and other chemotherapeutic drugs. Cancer Cell 11: 498–512 17560332 10.1016/j.ccr.2007.04.011

[bib33] Swanton C, Szallasi Z, Brenton JD, Downward J (2008) Functional genomic analysis of drug sensitivity pathways to guide adjuvant strategies in breast cancer. Breast Cancer Res 10: 214 18986507 10.1186/bcr2159 PMC2614525

[bib34] Swanton C, Tomlinson I, Downward J (2006) Chromosomal instability, colorectal cancer and taxane resistance. Cell Cycle 5: 818–823 16628000 10.4161/cc.5.8.2682

[bib35] Teschendorff AE, Miremadi A, Pinder SE, Ellis IO, Caldas C (2007) An immune response gene expression module identifies a good prognosis subtype in estrogen receptor negative breast cancer. Genome Biol 8: R157 17683518 10.1186/gb-2007-8-8-r157 PMC2374988

[bib36] Upender MB, Habermann JK, McShane LM, Korn EL, Barrett JC, Difilippantonio MJ, Ried T (2004) Chromosome transfer induced aneuploidy results in complex dysregulation of the cellular transcriptome in immortalized and cancer cells. Cancer Res 64: 6941–6949 15466185 10.1158/0008-5472.CAN-04-0474 PMC4772432

[bib37] van't Veer LJ, Bernards R (2008) Enabling personalized cancer medicine through analysis of gene-expression patterns. Nature 452: 564–570 18385730 10.1038/nature06915

[bib38] Walther A, Houlston R, Tomlinson I (2008) Association between chromosomal instability and prognosis in colorectal cancer: a meta-analysis. Gut 57: 941–950 18364437 10.1136/gut.2007.135004

[bib39] Yu SL, Chen HY, Chang GC, Chen CY, Chen HW, Singh S, Cheng CL, Yu CJ, Lee YC, Chen HS, Su TJ, Chiang CC, Li HN, Hong QS, Su HY, Chen CC, Chen WJ, Liu CC, Chan WK, Chen WJ, Li KC, Chen JJ, Yang PC (2008) MicroRNA signature predicts survival and relapse in lung cancer. Cancer Cell 13: 48–57 18167339 10.1016/j.ccr.2007.12.008

